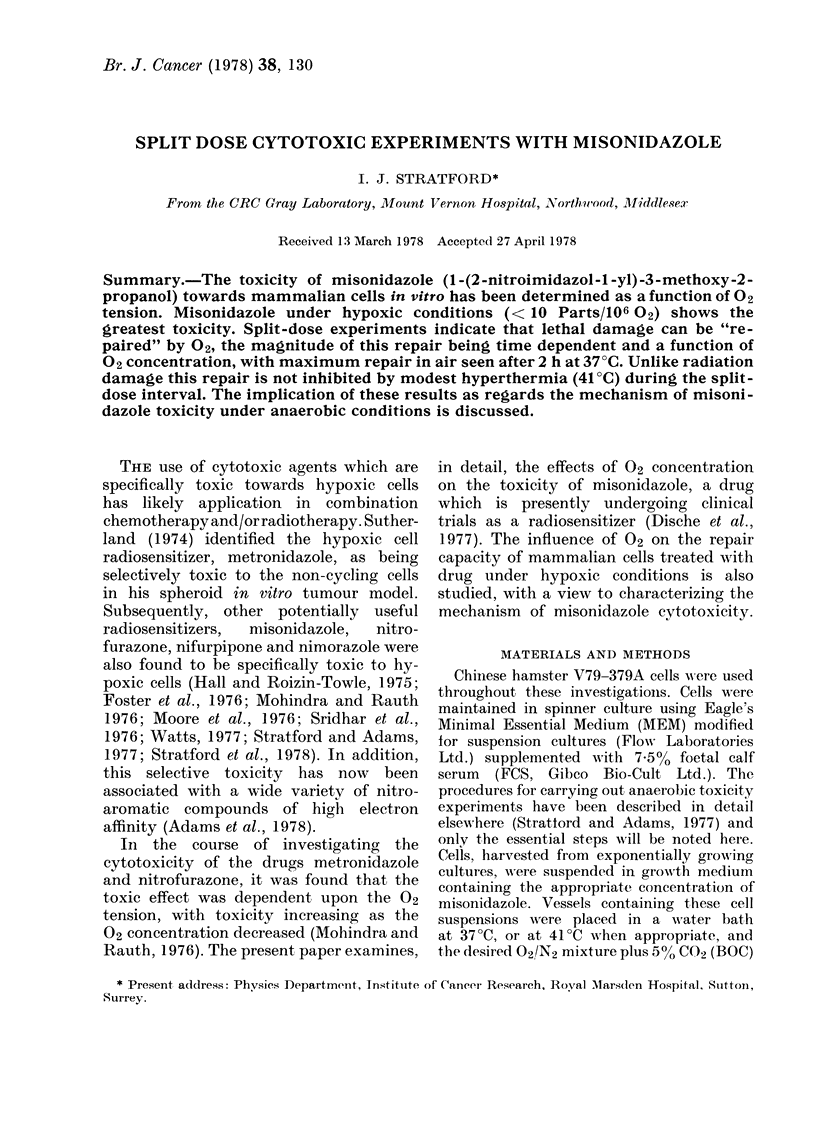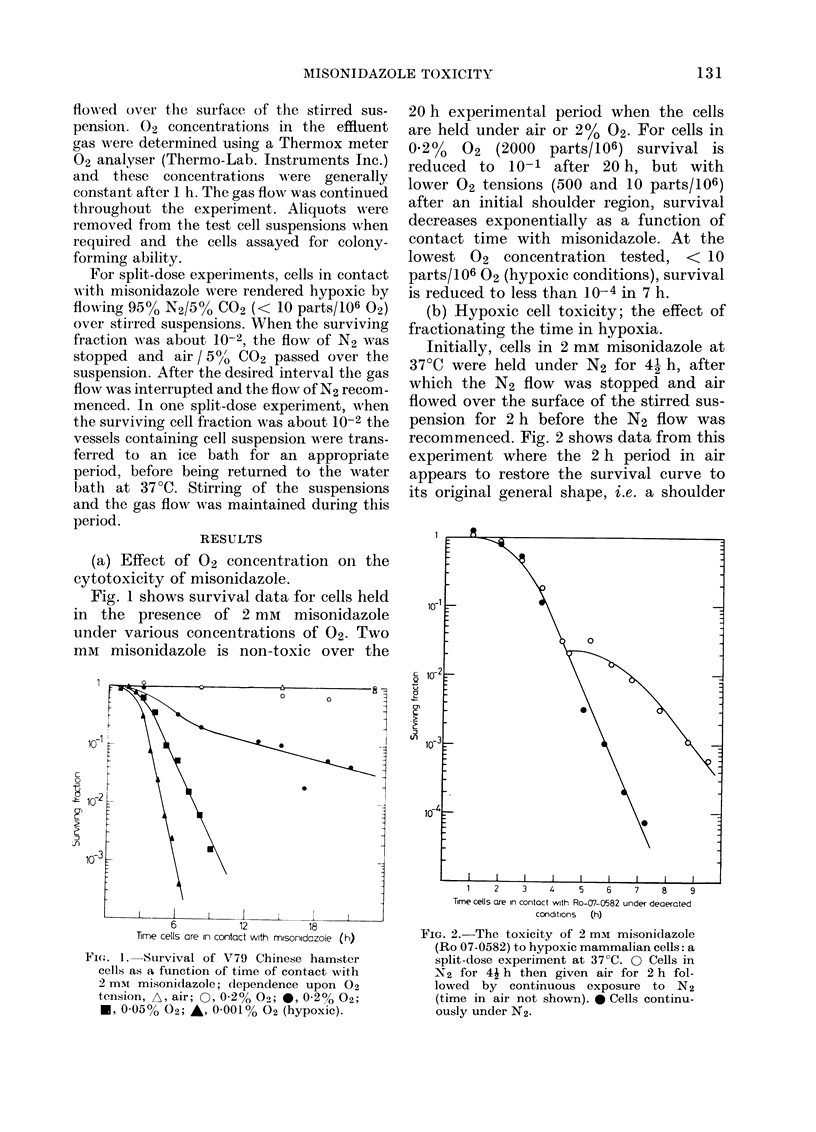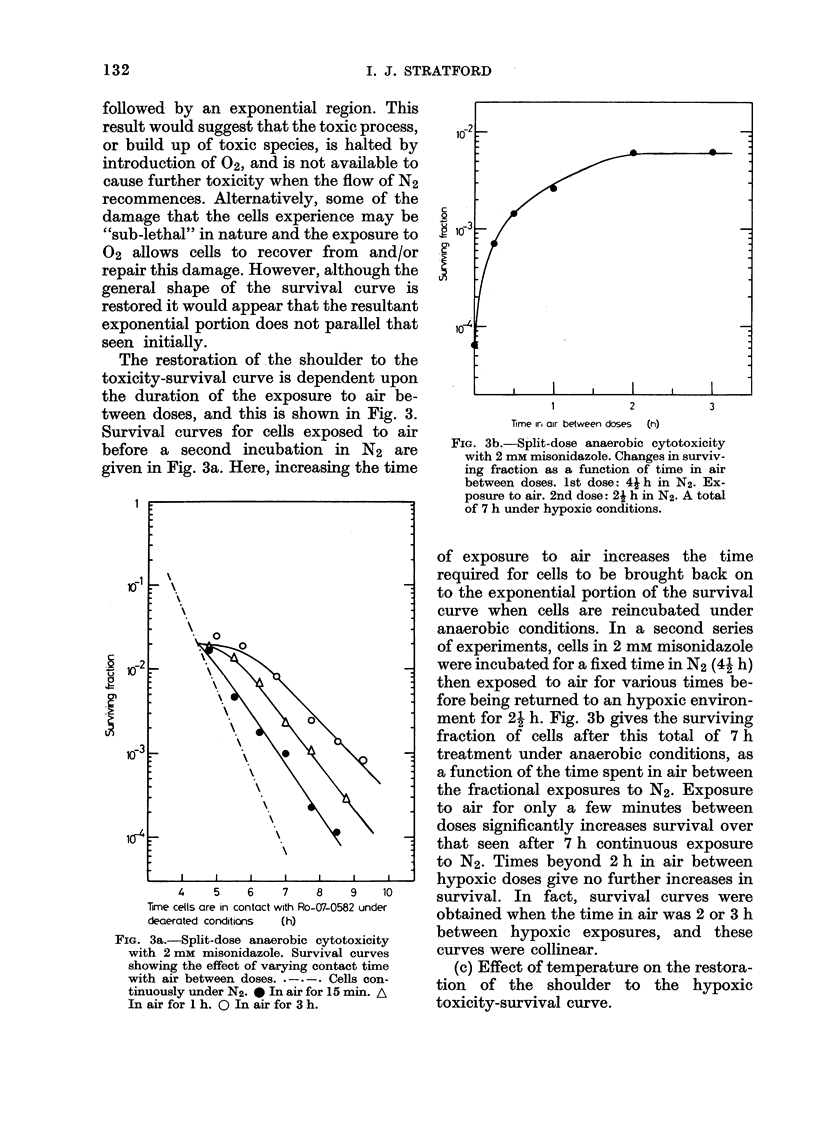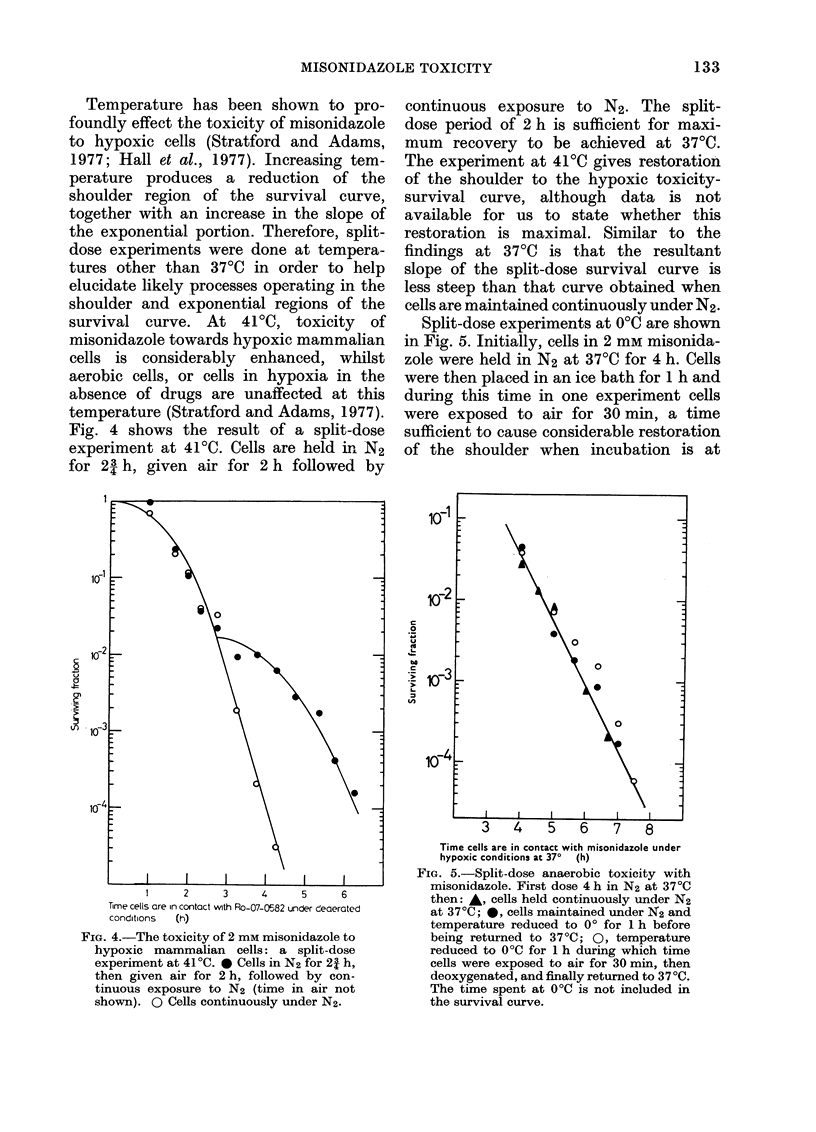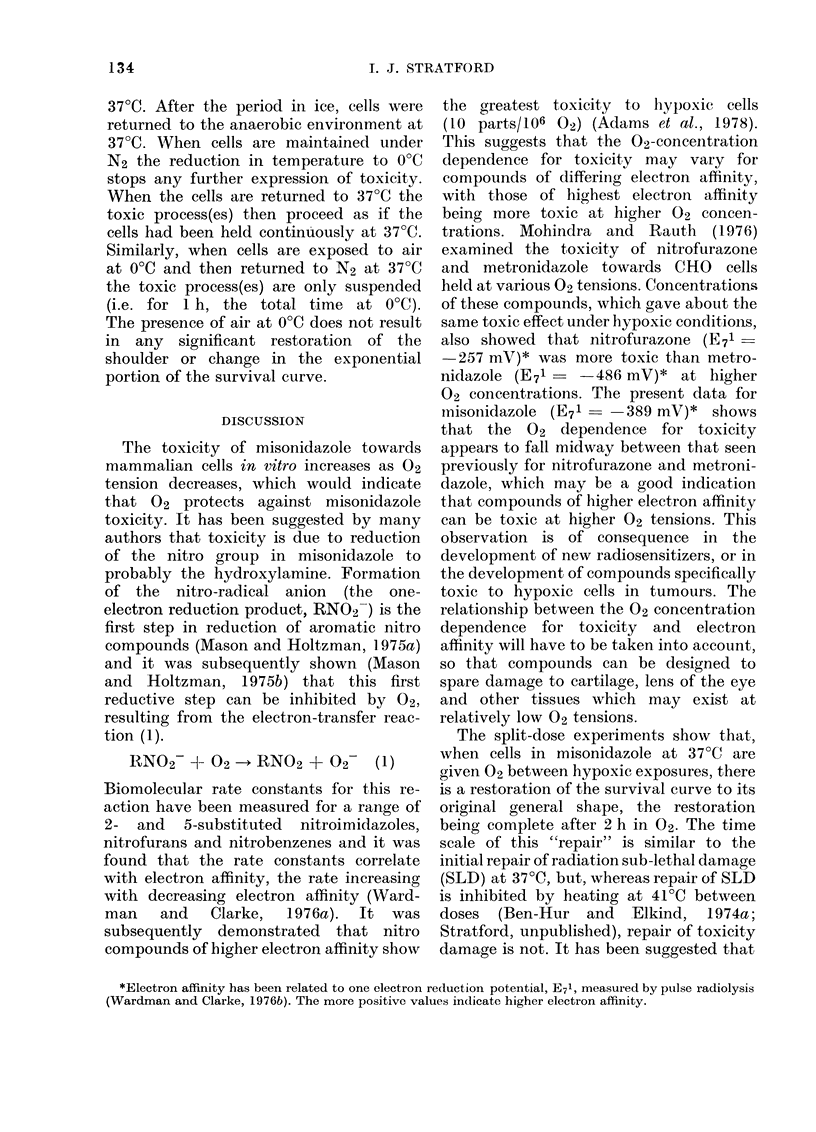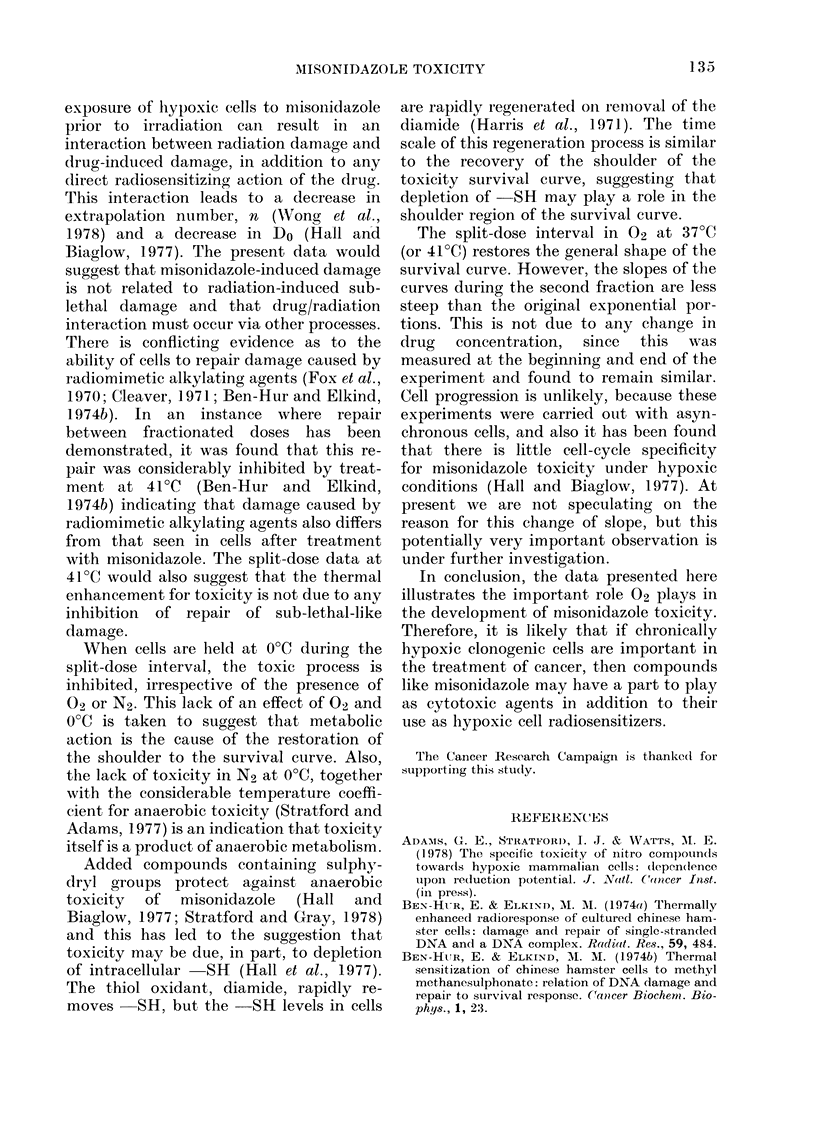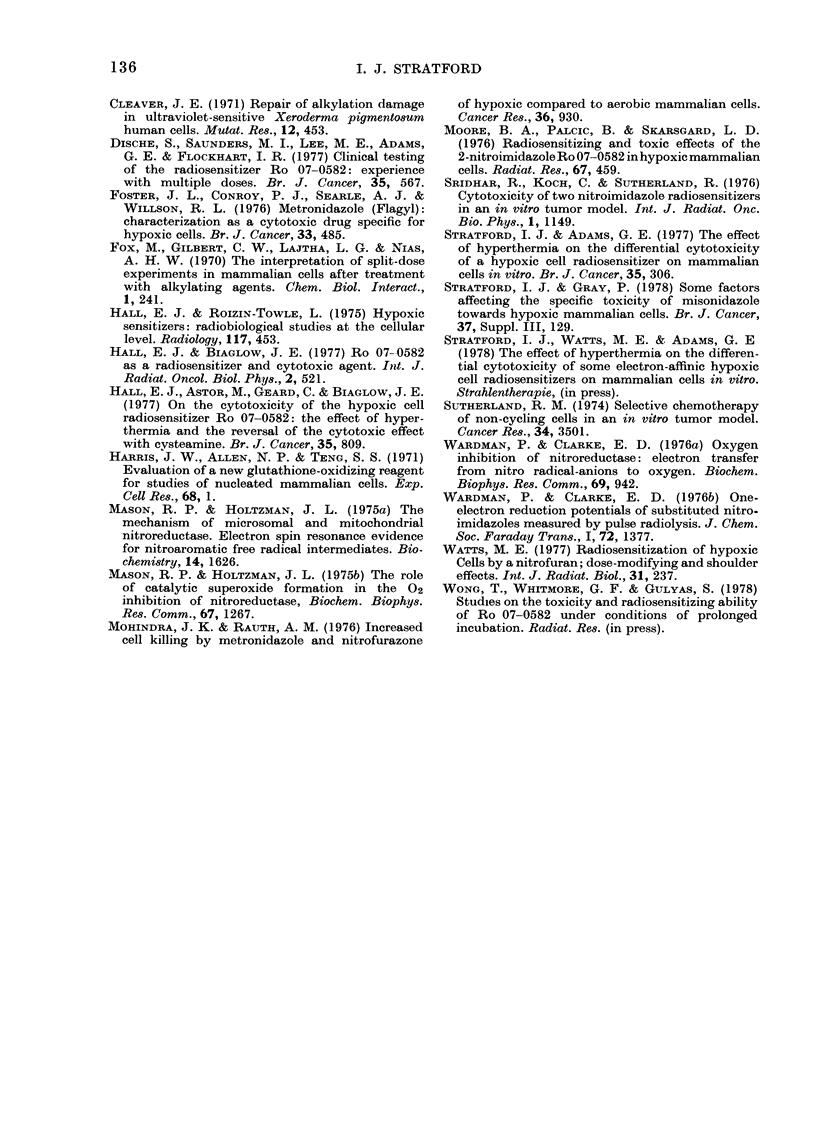# Split dose cytotoxic experiments with misonidazole.

**DOI:** 10.1038/bjc.1978.172

**Published:** 1978-07

**Authors:** I. J. Stratford

## Abstract

The toxicity of misonidazole (1-(2-nitroimidazol-1-yl)-3-methoxy-2-propanol) towards mammalian cells in vitro has been determined as a function of O2 tension. Misonidazole under hypoxic conditions (less than 10 Parts/10(6) O2) shows the greatest toxicity. Split-dose experiments indicate that lethal damage can be "repaired" by O2, the magnitude of this repair being time dependent and a function of O2 concentration, with maximum repair in air seen after 2 h at 37 degree C. Unlike radiation damage this repair is not inhibited by modest hyperthermia (41 degrees C) during the split-dose interval. The implication of these results as regards the mechanism of misonidazole toxicity under anaerobic conditions is discussed.


*Br. J. Cancer* (1978) **38**, 130

### SPLIT DOSE CYTOTOXIC EXPERIMENTS WITH MISONIDAZOLE

I. J. STRATFORD*

*From the CRC Gray Laboratory, Mount Vernon Hospital, Northwood, Middlesex*

Received 13 March 1978 Accepted 27 April 1978

**Summary.—The toxicity of misonidazole (1-(2-nitroimidazol-1-yl)-3-methoxy-2-propanol) towards mammalian cells *in vitro* has been determined as a function of $O_2$ tension. Misonidazole under hypoxic conditions ($<10$ Parts/$10^6$ $O_2$) shows the greatest toxicity. Split-dose experiments indicate that lethal damage can be "repaired" by $O_2$, the magnitude of this repair being time dependent and a function of $O_2$ concentration, with maximum repair in air seen after 2 h at 37°C. Unlike radiation damage this repair is not inhibited by modest hyperthermia (41°C) during the split-dose interval. The implication of these results as regards the mechanism of misonidazole toxicity under anaerobic conditions is discussed.**

THE use of cytotoxic agents which are specifically toxic towards hypoxic cells has likely application in combination chemotherapy and/or radiotherapy. Sutherland (1974) identified the hypoxic cell radiosensitizer, metronidazole, as being selectively toxic to the non-cycling cells in his spheroid *in vitro* tumour model. Subsequently, other potentially useful radiosensitizers, misonidazole, nitrofurazone, nifurpipone and nimorazole were also found to be specifically toxic to hypoxic cells (Hall and Roizin-Towle, 1975; Foster *et al.*, 1976; Mohindra and Rauth 1976; Moore *et al.*, 1976; Sridhar *et al.*, 1976; Watts, 1977; Stratford and Adams, 1977; Stratford *et al.*, 1978). In addition, this selective toxicity has now been associated with a wide variety of nitroaromatic compounds of high electron affinity (Adams *et al.*, 1978).

In the course of investigating the cytotoxicity of the drugs metronidazole and nitrofurazone, it was found that the toxic effect was dependent upon the $O_2$ tension, with toxicity increasing as the $O_2$ concentration decreased (Mohindra and Rauth, 1976). The present paper examines, in detail, the effects of $O_2$ concentration on the toxicity of misonidazole, a drug which is presently undergoing clinical trials as a radiosensitizer (Dische *et al.*, 1977). The influence of $O_2$ on the repair capacity of mammalian cells treated with drug under hypoxic conditions is also studied, with a view to characterizing the mechanism of misonidazole cytotoxicity.

### MATERIALS AND METHODS

Chinese hamster V79–379A cells were used throughout these investigations. Cells were maintained in spinner culture using Eagle's Minimal Essential Medium (MEM) modified for suspension cultures (Flow Laboratories Ltd.) supplemented with 7·5% foetal calf serum (FCS, Gibco Bio-Cult Ltd.). The procedures for carrying out anaerobic toxicity experiments have been described in detail elsewhere (Stratford and Adams, 1977) and only the essential steps will be noted here. Cells, harvested from exponentially growing cultures, were suspended in growth medium containing the appropriate concentration of misonidazole. Vessels containing these cell suspensions were placed in a water bath at 37°C, or at 41°C when appropriate, and the desired $O_2/N_2$ mixture plus 5% $CO_2$ (BOC)

* Present address: Physics Department, Institute of Cancer Research, Royal Marsden Hospital, Sutton, Surrey.

flowed over the surface of the stirred suspension. $O_2$ concentrations in the effluent gas were determined using a Thermox meter $O_2$ analyser (Thermo-Lab. Instruments Inc.) and these concentrations were generally constant after 1 h. The gas flow was continued throughout the experiment. Aliquots were removed from the test cell suspensions when required and the cells assayed for colony-forming ability.

For split-dose experiments, cells in contact with misonidazole were rendered hypoxic by flowing 95% $N_2$/5% $CO_2$ (< 10 parts/$10^6$ $O_2$) over stirred suspensions. When the surviving fraction was about $10^{-2}$, the flow of $N_2$ was stopped and air / 5% $CO_2$ passed over the suspension. After the desired interval the gas flow was interrupted and the flow of $N_2$ recommenced. In one split-dose experiment, when the surviving cell fraction was about $10^{-2}$ the vessels containing cell suspension were transferred to an ice bath for an appropriate period, before being returned to the water bath at 37°C. Stirring of the suspensions and the gas flow was maintained during this period.

### RESULTS

(a) Effect of $O_2$ concentration on the cytotoxicity of misonidazole.

Fig. 1 shows survival data for cells held in the presence of 2 mM misonidazole under various concentrations of $O_2$. Two mM misonidazole is non-toxic over the

FIG. 1.—Survival of V79 Chinese hamster cells as a function of time of contact with 2 mM misonidazole; dependence upon $O_2$ tension, △, air; ○, 0·2% $O_2$; ●, 0·2% $O_2$; ■, 0·05% $O_2$; ▲, 0·001% $O_2$ (hypoxic).

20 h experimental period when the cells are held under air or 2% $O_2$. For cells in 0·2% $O_2$ (2000 parts/$10^6$) survival is reduced to $10^{-1}$ after 20 h, but with lower $O_2$ tensions (500 and 10 parts/$10^6$) after an initial shoulder region, survival decreases exponentially as a function of contact time with misonidazole. At the lowest $O_2$ concentration tested, < 10 parts/$10^6$ $O_2$ (hypoxic conditions), survival is reduced to less than $10^{-4}$ in 7 h.

(b) Hypoxic cell toxicity; the effect of fractionating the time in hypoxia.

Initially, cells in 2 mM misonidazole at 37°C were held under $N_2$ for 4½ h, after which the $N_2$ flow was stopped and air flowed over the surface of the stirred suspension for 2 h before the $N_2$ flow was recommenced. Fig. 2 shows data from this experiment where the 2 h period in air appears to restore the survival curve to its original general shape, *i.e.* a shoulder

FIG. 2.—The toxicity of 2 mM misonidazole (Ro 07-0582) to hypoxic mammalian cells: a split-dose experiment at 37°C. ○ Cells in $N_2$ for 4½ h then given air for 2 h followed by continuous exposure to $N_2$ (time in air not shown). ● Cells continuously under $N_2$.

followed by an exponential region. This result would suggest that the toxic process, or build up of toxic species, is halted by introduction of $O_2$, and is not available to cause further toxicity when the flow of $N_2$ recommences. Alternatively, some of the damage that the cells experience may be "sub-lethal" in nature and the exposure to $O_2$ allows cells to recover from and/or repair this damage. However, although the general shape of the survival curve is restored it would appear that the resultant exponential portion does not parallel that seen initially.

The restoration of the shoulder to the toxicity-survival curve is dependent upon the duration of the exposure to air between doses, and this is shown in Fig. 3. Survival curves for cells exposed to air before a second incubation in $N_2$ are given in Fig. 3a. Here, increasing the time

FIG. 3a.—Split-dose anaerobic cytotoxicity with 2 mM misonidazole. Survival curves showing the effect of varying contact time with air between doses. .—.—. Cells continuously under $N_2$. ● In air for 15 min. △ In air for 1 h. ○ In air for 3 h.

FIG. 3b.—Split-dose anaerobic cytotoxicity with 2 mM misonidazole. Changes in surviving fraction as a function of time in air between doses. 1st dose: 4½ h in $N_2$. Exposure to air. 2nd dose: 2½ h in $N_2$. A total of 7 h under hypoxic conditions.

of exposure to air increases the time required for cells to be brought back on to the exponential portion of the survival curve when cells are reincubated under anaerobic conditions. In a second series of experiments, cells in 2 mM misonidazole were incubated for a fixed time in $N_2$ (4½ h) then exposed to air for various times before being returned to an hypoxic environment for 2½ h. Fig. 3b gives the surviving fraction of cells after this total of 7 h treatment under anaerobic conditions, as a function of the time spent in air between the fractional exposures to $N_2$. Exposure to air for only a few minutes between doses significantly increases survival over that seen after 7 h continuous exposure to $N_2$. Times beyond 2 h in air between hypoxic doses give no further increases in survival. In fact, survival curves were obtained when the time in air was 2 or 3 h between hypoxic exposures, and these curves were collinear.

(c) Effect of temperature on the restoration of the shoulder to the hypoxic toxicity-survival curve.

Temperature has been shown to profoundly effect the toxicity of misonidazole to hypoxic cells (Stratford and Adams, 1977; Hall *et al.*, 1977). Increasing temperature produces a reduction of the shoulder region of the survival curve, together with an increase in the slope of the exponential portion. Therefore, split-dose experiments were done at temperatures other than 37°C in order to help elucidate likely processes operating in the shoulder and exponential regions of the survival curve. At 41°C, toxicity of misonidazole towards hypoxic mammalian cells is considerably enhanced, whilst aerobic cells, or cells in hypoxia in the absence of drugs are unaffected at this temperature (Stratford and Adams, 1977). Fig. 4 shows the result of a split-dose experiment at 41°C. Cells are held in $N_2$ for $2\frac{3}{4}$ h, given air for 2 h followed by continuous exposure to $N_2$. The split-dose period of 2 h is sufficient for maximum recovery to be achieved at 37°C. The experiment at 41°C gives restoration of the shoulder to the hypoxic toxicity-survival curve, although data is not available for us to state whether this restoration is maximal. Similar to the findings at 37°C is that the resultant slope of the split-dose survival curve is less steep than that curve obtained when cells are maintained continuously under $N_2$.

Split-dose experiments at 0°C are shown in Fig. 5. Initially, cells in 2 mM misonidazole were held in $N_2$ at 37°C for 4 h. Cells were then placed in an ice bath for 1 h and during this time in one experiment cells were exposed to air for 30 min, a time sufficient to cause considerable restoration of the shoulder when incubation is at

FIG. 4.—The toxicity of 2 mM misonidazole to hypoxic mammalian cells: a split-dose experiment at 41°C. ● Cells in $N_2$ for $2\frac{3}{4}$ h, then given air for 2 h, followed by continuous exposure to $N_2$ (time in air not shown). ○ Cells continuously under $N_2$.

FIG. 5.—Split-dose anaerobic toxicity with misonidazole. First dose 4 h in $N_2$ at 37°C then: ▲, cells held continuously under $N_2$ at 37°C; ●, cells maintained under $N_2$ and temperature reduced to 0° for 1 h before being returned to 37°C; ○, temperature reduced to 0°C for 1 h during which time cells were exposed to air for 30 min, then deoxygenated, and finally returned to 37°C. The time spent at 0°C is not included in the survival curve.

37°C. After the period in ice, cells were returned to the anaerobic environment at 37°C. When cells are maintained under $N_2$ the reduction in temperature to 0°C stops any further expression of toxicity. When the cells are returned to 37°C the toxic process(es) then proceed as if the cells had been held continuously at 37°C. Similarly, when cells are exposed to air at 0°C and then returned to $N_2$ at 37°C the toxic process(es) are only suspended (i.e. for 1 h, the total time at 0°C). The presence of air at 0°C does not result in any significant restoration of the shoulder or change in the exponential portion of the survival curve.

### DISCUSSION

The toxicity of misonidazole towards mammalian cells *in vitro* increases as $O_2$ tension decreases, which would indicate that $O_2$ protects against misonidazole toxicity. It has been suggested by many authors that toxicity is due to reduction of the nitro group in misonidazole to probably the hydroxylamine. Formation of the nitro-radical anion (the one-electron reduction product, $RNO_2^-$) is the first step in reduction of aromatic nitro compounds (Mason and Holtzman, 1975*a*) and it was subsequently shown (Mason and Holtzman, 1975*b*) that this first reductive step can be inhibited by $O_2$, resulting from the electron-transfer reaction (1).

$$RNO_2^- + O_2 \rightarrow RNO_2 + O_2^- \quad (1)$$

Biomolecular rate constants for this reaction have been measured for a range of 2- and 5-substituted nitroimidazoles, nitrofurans and nitrobenzenes and it was found that the rate constants correlate with electron affinity, the rate increasing with decreasing electron affinity (Wardman and Clarke, 1976*a*). It was subsequently demonstrated that nitro compounds of higher electron affinity show the greatest toxicity to hypoxic cells (10 parts/$10^6$ $O_2$) (Adams *et al.*, 1978). This suggests that the $O_2$-concentration dependence for toxicity may vary for compounds of differing electron affinity, with those of highest electron affinity being more toxic at higher $O_2$ concentrations. Mohindra and Rauth (1976) examined the toxicity of nitrofurazone and metronidazole towards CHO cells held at various $O_2$ tensions. Concentrations of these compounds, which gave about the same toxic effect under hypoxic conditions, also showed that nitrofurazone ($E_7^1 = -257$ mV)* was more toxic than metronidazole ($E_7^1 = -486$ mV)* at higher $O_2$ concentrations. The present data for misonidazole ($E_7^1 = -389$ mV)* shows that the $O_2$ dependence for toxicity appears to fall midway between that seen previously for nitrofurazone and metronidazole, which may be a good indication that compounds of higher electron affinity can be toxic at higher $O_2$ tensions. This observation is of consequence in the development of new radiosensitizers, or in the development of compounds specifically toxic to hypoxic cells in tumours. The relationship between the $O_2$ concentration dependence for toxicity and electron affinity will have to be taken into account, so that compounds can be designed to spare damage to cartilage, lens of the eye and other tissues which may exist at relatively low $O_2$ tensions.

The split-dose experiments show that, when cells in misonidazole at 37°C are given $O_2$ between hypoxic exposures, there is a restoration of the survival curve to its original general shape, the restoration being complete after 2 h in $O_2$. The time scale of this "repair" is similar to the initial repair of radiation sub-lethal damage (SLD) at 37°C, but, whereas repair of SLD is inhibited by heating at 41°C between doses (Ben-Hur and Elkind, 1974*a*; Stratford, unpublished), repair of toxicity damage is not. It has been suggested that

*Electron affinity has been related to one electron reduction potential, $E_7^1$, measured by pulse radiolysis (Wardman and Clarke, 1976*b*). The more positive values indicate higher electron affinity.

exposure of hypoxic cells to misonidazole prior to irradiation can result in an interaction between radiation damage and drug-induced damage, in addition to any direct radiosensitizing action of the drug. This interaction leads to a decrease in extrapolation number, $n$ (Wong *et al.*, 1978) and a decrease in $D_0$ (Hall and Biaglow, 1977). The present data would suggest that misonidazole-induced damage is not related to radiation-induced sub-lethal damage and that drug/radiation interaction must occur via other processes. There is conflicting evidence as to the ability of cells to repair damage caused by radiomimetic alkylating agents (Fox *et al.*, 1970; Cleaver, 1971; Ben-Hur and Elkind, 1974*b*). In an instance where repair between fractionated doses has been demonstrated, it was found that this repair was considerably inhibited by treatment at 41°C (Ben-Hur and Elkind, 1974*b*) indicating that damage caused by radiomimetic alkylating agents also differs from that seen in cells after treatment with misonidazole. The split-dose data at 41°C would also suggest that the thermal enhancement for toxicity is not due to any inhibition of repair of sub-lethal-like damage.

When cells are held at 0°C during the split-dose interval, the toxic process is inhibited, irrespective of the presence of $O_2$ or $N_2$. This lack of an effect of $O_2$ and 0°C is taken to suggest that metabolic action is the cause of the restoration of the shoulder to the survival curve. Also, the lack of toxicity in $N_2$ at 0°C, together with the considerable temperature coefficient for anaerobic toxicity (Stratford and Adams, 1977) is an indication that toxicity itself is a product of anaerobic metabolism.

Added compounds containing sulphydryl groups protect against anaerobic toxicity of misonidazole (Hall and Biaglow, 1977; Stratford and Gray, 1978) and this has led to the suggestion that toxicity may be due, in part, to depletion of intracellular —SH (Hall *et al.*, 1977). The thiol oxidant, diamide, rapidly removes —SH, but the —SH levels in cells are rapidly regenerated on removal of the diamide (Harris *et al.*, 1971). The time scale of this regeneration process is similar to the recovery of the shoulder of the toxicity survival curve, suggesting that depletion of —SH may play a role in the shoulder region of the survival curve.

The split-dose interval in $O_2$ at 37°C (or 41°C) restores the general shape of the survival curve. However, the slopes of the curves during the second fraction are less steep than the original exponential portions. This is not due to any change in drug concentration, since this was measured at the beginning and end of the experiment and found to remain similar. Cell progression is unlikely, because these experiments were carried out with asynchronous cells, and also it has been found that there is little cell-cycle specificity for misonidazole toxicity under hypoxic conditions (Hall and Biaglow, 1977). At present we are not speculating on the reason for this change of slope, but this potentially very important observation is under further investigation.

In conclusion, the data presented here illustrates the important role $O_2$ plays in the development of misonidazole toxicity. Therefore, it is likely that if chronically hypoxic clonogenic cells are important in the treatment of cancer, then compounds like misonidazole may have a part to play as cytotoxic agents in addition to their use as hypoxic cell radiosensitizers.

The Cancer Research Campaign is thanked for supporting this study.

### REFERENCES

ADAMS, G. E., STRATFORD, I. J. & WATTS, M. E. (1978) The specific toxicity of nitro compounds towards hypoxic mammalian cells: dependence upon reduction potential. *J. Natl. Cancer Inst.* (in press).

BEN-HUR, E. & ELKIND, M. M. (1974*a*) Thermally enhanced radioresponse of cultured chinese hamster cells: damage and repair of single-stranded DNA and a DNA complex. *Radiat. Res.*, **59**, 484.

BEN-HUR, E. & ELKIND, M. M. (1974*b*) Thermal sensitization of chinese hamster cells to methyl methanesulphonate: relation of DNA damage and repair to survival response. *Cancer Biochem. Biophys.*, **1**, 23.

CLEAVER, J. E. (1971) Repair of alkylation damage in ultraviolet-sensitive *Xeroderma pigmentosum* human cells. *Mutat. Res.*, **12**, 453.

DISCHE, S., SAUNDERS, M. I., LEE, M. E., ADAMS, G. E. & FLOCKHART, I. R. (1977) Clinical testing of the radiosensitizer Ro 07-0582: experience with multiple doses. *Br. J. Cancer*, **35**, 567.

FOSTER, J. L., CONROY, P. J., SEARLE, A. J. & WILLSON, R. L. (1976) Metronidazole (Flagyl): characterization as a cytotoxic drug specific for hypoxic cells. *Br. J. Cancer*, **33**, 485.

FOX, M., GILBERT, C. W., LAJTHA, L. G. & NIAS, A. H. W. (1970) The interpretation of split-dose experiments in mammalian cells after treatment with alkylating agents. *Chem. Biol. Interact.*, **1**, 241.

HALL, E. J. & ROIZIN-TOWLE, L. (1975) Hypoxic sensitizers: radiobiological studies at the cellular level. *Radiology*, **117**, 453.

HALL, E. J. & BIAGLOW, J. E. (1977) Ro 07-0582 as a radiosensitizer and cytotoxic agent. *Int. J. Radiat. Oncol. Biol. Phys.*, **2**, 521.

HALL, E. J., ASTOR, M., GEARD, C. & BIAGLOW, J. E. (1977) On the cytotoxicity of the hypoxic cell radiosensitizer Ro 07-0582: the effect of hyperthermia and the reversal of the cytotoxic effect with cysteamine. *Br. J. Cancer*, **35**, 809.

HARRIS, J. W., ALLEN, N. P. & TENG, S. S. (1971) Evaluation of a new glutathione-oxidizing reagent for studies of nucleated mammalian cells. *Exp. Cell Res.*, **68**, 1.

MASON, R. P. & HOLTZMAN, J. L. (1975*a*) The mechanism of microsomal and mitochondrial nitroreductase. Electron spin resonance evidence for nitroaromatic free radical intermediates. *Biochemistry*, **14**, 1626.

MASON, R. P. & HOLTZMAN, J. L. (1975*b*) The role of catalytic superoxide formation in the $O_2$ inhibition of nitroreductase, *Biochem. Biophys. Res. Comm.*, **67**, 1267.

MOHINDRA, J. K. & RAUTH, A. M. (1976) Increased cell killing by metronidazole and nitrofurazone of hypoxic compared to aerobic mammalian cells. *Cancer Res.*, **36**, 930.

MOORE, B. A., PALCIC, B. & SKARSGARD, L. D. (1976) Radiosensitizing and toxic effects of the 2-nitroimidazole Ro 07-0582 in hypoxic mammalian cells. *Radiat. Res.*, **67**, 459.

SRIDHAR, R., KOCH, C. & SUTHERLAND, R. (1976) Cytotoxicity of two nitroimidazole radiosensitizers in an *in vitro* tumor model. *Int. J. Radiat. Onc. Bio. Phys.*, **1**, 1149.

STRATFORD, I. J. & ADAMS, G. E. (1977) The effect of hyperthermia on the differential cytotoxicity of a hypoxic cell radiosensitizer on mammalian cells *in vitro*. *Br. J. Cancer*, **35**, 306.

STRATFORD, I. J. & GRAY, P. (1978) Some factors affecting the specific toxicity of misonidazole towards hypoxic mammalian cells. *Br. J. Cancer*, **37**, Suppl. III, 129.

STRATFORD, I. J., WATTS, M. E. & ADAMS, G. E (1978) The effect of hyperthermia on the differential cytotoxicity of some electron-affinic hypoxic cell radiosensitizers on mammalian cells *in vitro*. *Strahlentherapie*, (in press).

SUTHERLAND, R. M. (1974) Selective chemotherapy of non-cycling cells in an *in vitro* tumor model. *Cancer Res.*, **34**, 3501.

WARDMAN, P. & CLARKE, E. D. (1976*a*) Oxygen inhibition of nitroreductase: electron transfer from nitro radical-anions to oxygen. *Biochem. Biophys. Res. Comm.*, **69**, 942.

WARDMAN, P. & CLARKE, E. D. (1976*b*) One-electron reduction potentials of substituted nitroimidazoles measured by pulse radiolysis. *J. Chem. Soc. Faraday Trans.*, I, **72**, 1377.

WATTS, M. E. (1977) Radiosensitization of hypoxic Cells by a nitrofuran; dose-modifying and shoulder effects. *Int. J. Radiat. Biol.*, **31**, 237.

WONG, T., WHITMORE, G. F. & GULYAS, S. (1978) Studies on the toxicity and radiosensitizing ability of Ro 07-0582 under conditions of prolonged incubation. *Radiat. Res.* (in press).